# Knowledge of Droplet Infection and Airborne Isolation Among Dental Students in South India: A Questionnaire Study

**DOI:** 10.7759/cureus.41130

**Published:** 2023-06-29

**Authors:** Bellamkonda Pavani, Anitha Akkaloori, S Gauri Chandran, S.S. Malarvizhe, M Gomathy, Srinivasan Vinodhini

**Affiliations:** 1 Public Health Dentistry, Sathyabama Dental College and Hospital, Chennai, IND; 2 Public Health Dentistry, Government Dental College and Hospital, Hyderabad, IND; 3 Dentistry, Sathyabama Dental College and Hospital, Chennai, IND

**Keywords:** droplet, airborne, universal precautions, south india, dental students, infection control

## Abstract

Background

Infections can spread within the dental clinic through many routes. Interrupting this transmission of infection within the dental office is an important part of the dental practice. This study aimed to assess the knowledge, attitude, and practice regarding precautions against droplet and airborne infections among dental students in South India.

Methodology

A cross-sectional, questionnaire-based survey was conducted among 236 undergraduate dental students in Chennai. The questionnaire consisted of 11 questions in three categories of knowledge, attitude, and practice regarding airborne and droplet isolation precautions. The collected data were compiled and analyzed using SPSS version 13 software (SPSS Inc., Chicago, IL, USA).

Results

The frequency scores of knowledge, attitude, and practice regarding droplet and airborne isolation precautions showed that dental students were very much aware of the precautions and the guidelines. The mean scores for knowledge, attitude, and practice were 7.70 ± 2.48, 37.22 ± 6.98, and 7.1 ± 1.64, respectively. There were no significant differences among subgroups (third-year students, fourth-year students, and interns) regarding knowledge, attitude, and practice. A positive linear correlation was observed between knowledge and attitude, knowledge and practice, and attitude and practice (p < 0.05).

Conclusions

According to the results of this study, dental students had adequate knowledge, positive attitudes, and compliance. Training programs on isolation precautions are needed to sustain and update the knowledge according to the changing trends in infectious diseases.

## Introduction

Transmission of infectious diseases occurs frequently in the dental setting due to the aerosolization potential of blood and saliva during dental procedures. Interrupting the transmission of infection within the dental office is an important part of the dental practice, and the dental workforce should follow basic infection control measures while treating patients [1]. Infections can spread within the dental operatory through many routes, such as direct contact with blood, oral, and other body fluids; contact with contaminated dental equipment, instruments, and surfaces; and through airborne contaminants present in droplets or spray of oral or nasal fluids [2,3].

Dental professionals are more prone to contracting dangerous infections such as tuberculosis, viral hepatitis, staphylococci, streptococci, herpes simplex virus, and human immunodeficiency virus (HIV) [1]. A recent addition to this list of infections is COVID-19. This disease caused by coronavirus has become a pandemic affecting the entire world in a relatively short duration. The transmission of COVID-19 occurs mainly through the respiratory system [4,5]. Dental practitioners faced unprecedented challenges during COVID-19, as it was a highly infectious disease [6,7].

Although few studies have investigated the transmission of infections in dental practice, indirect evidence indicates that antibodies to several viruses found in saliva are relatively more common in dental practitioners than in the general population [8,9].

The increasing incidence of infection transmission has led to increased awareness regarding the risk of cross-infection between dentists and patients. This rise in awareness has led to a resurgence of infection control measures in recent years [10].

Dental colleges play a key role in training dental students by providing them with adequate knowledge and ensuring strict adherence to infection control guidelines. Even though significant importance is given to infection control protocols during training, very few dentists practice these protocols in their clinics [11-13]. Several studies have reported a lack of compliance among dental students in practicing the protocols [9,12,14-16].

With this background, this study aimed to assess the knowledge, attitudes, and practice toward droplet and airborne isolation precautions among dental students in Chennai, India. The study also analyzed if any correlation exists between the knowledge, attitude, and practice scores.

## Materials and methods

A questionnaire-based survey was conducted at Sathyabama Dental College and Hospital in Chennai, India among 250 students (the total number of students who were enrolled in the third year, fourth year, and internship of the undergraduate dentistry course). Out of the 250 questionnaire forms distributed to various respondents, 236 forms were received. In total, 72 respondents were third-year dental students, 86 were fourth-year students, and 78 were interns.

The questionnaire was adapted from a similar study reported by Askarian et al. [12]. The questionnaire comprised 11 closed-ended questions with a separate set of responses for knowledge, attitude, and practice of the guidelines regarding droplet and airborne isolation precautions, as illustrated by the Centres for Disease Control and Prevention (CDC). Three separate sets of responses corresponding to knowledge, attitude, and practice were given under each question. The validity and reliability of the questionnaire were tested on a sample of students to ensure clarity, feasibility, and interpretation. The knowledge was assessed using three responses, namely, yes, no, and I do not know. One point was allotted when the answer to the question agreed with the CDC guidelines. Thus, the total score for knowledge could range from 0 to 11; 0 if all the answers were incorrect, and 11 if all the answers were correct.

The questions that assessed attitude had five responses (very high, high, intermediate, low, and no importance which reflected their attitude as very strong, strong, considerable, weak, or null, respectively). The response very high (corresponding to very strong attitude) was given 4 points while no importance (corresponding to null attitude) received zero points. Thus, the total score could range from 0 (if all responses were null) to 44 (if all responses were very strong) based on the response chosen.

Cronbach’s alpha was used to assess the validity of the questionnaire, which was found to be 0.81. Categorical variables were compared using the chi-square test. Analysis of variance was used to compare the differences in the mean scores of knowledge, attitude, and practice between the three groups (third-year students, final-year students, and interns). Spearman’s correlation coefficient was used to assess the correlation between knowledge, attitude, and practice scores. P-values ≤0.05 were considered statistically significant. SPSS version 13 (SPSS Inc., Chicago, IL, USA) was used for data analysis.

## Results

The response rates of the dental students were 89.5%, 92.1%, and 92.4% among third-year students, fourth-year students, and interns respectively. Significant differences were not observed regarding the level of training (p = 0.235), gender (p = 0.356), and age (p = 0.432) (Table 1).

**Table 1:** Demographic characteristics of study participants. NS = not significant

Parameter	Third year	Fourth year	Interns
Gender			
Male	11 (15.3%)	9(10.5%)	13 (16.7%)
Female	61 (84.7%)	77 (89.5%)	65 (83.3%)
P = 0.356^NS^			
Mean age (in years)	21.2 ± 1.80	21.90 ± 1.67	23.46 ± 2.01
P = 0.432^NS^			
Additional training on infection control/isolation precautions			
Yes	27 (36.1)	32 (46.6%)	36 (46.2%)
No	46 (63.9%)	46 (53.4%)	42 (53.8%)
P = 0.235^NS^			

Table 2 shows that dental students were aware of the risks of airborne and droplet transmission and were applying this knowledge in their regular practice.

**Table 2:** Frequency distribution of answers regarding knowledge, attitude, and practice on droplet and airborne precautions.

Questions	Knowledge		Attitude		Practice	
	Correct, n (%)	Incorrect, n (%)	Correct, n (%)	Incorrect, n (%)	Correct, n (%)	Incorrect, n (%)
Patients with a droplet-spread disease should be isolated in a private room	221 (93.7)	15 (6.3)	198 (83.9)	38 (16.1)	112 (47.6)	124 (52.4)
Patients with a droplet-spread disease should be kept apart at a distance of at least 150 cm	159 (67.4)	77 (32.6)	180 (76.3)	56 (23.7)	86 (36.4)	150 (63.6)
Patients with a droplet-spread disease should wear a mask during transport	181 (76.8)	55 (23.2)	219 (92.6)	17 (7.4)	218 (92.4)	18 (7.6)
Masks should be worn if or when a subject is within a 90 cm distance from a patient under droplet precaution care	150 (63.8)	86 (36.2 )	181 (76.8)	55 (23.2)	160 (67.8)	76 (33.2)
Hospital wards should be notified before receiving a patient needing droplet precaution	160 (67.6)	76 (32.4)	220 (93.2)	16 (6.8)	152 (64.7)	84 (35.3)
Patients with an airborne transmissible disease should be isolated in a private room with negative pressure	147 (62.3)	89 (37.7)	150 (63.6)	86 (36.4)	130 (54.7)	106 (45.3)
The door of the patient’s room with an airborne transmissible disease should always be closed	151 (63.8)	85 (36.2)	213 (90.4)	23 (9.6)	139 (59.2)	97 (40.8)
Wearing a mask is necessary when entering the room of patients with chickenpox or measles	161 (68.4)	75 (31.6)	180 (76.5)	56 (23.5)	173 (73.4)	63 (26.6)
All healthcare workers should be vaccinated with the BCG vaccine	162 (68.7)	74 (31.3)	220 (93.4)	16 (6.6)	200 (84.6)	36 (15.4)
Wards should be notified before receiving a patient requiring airborne precautions	176 (74.8)	60 (25.2)	222 (94.2)	14 (5.8)	171 (72.7)	65 (27.3)
Patients requiring airborne precautions should wear a surgical mask when being transported	150 (63.4)	86 (36.6)	213 (90.4)	23 (9.6)	136 (57.7)	100 (42.3)

The mean scores were calculated based on the points given for correct responses, as mentioned earlier. The resulting mean scores for knowledge, attitude, and practice were 7.70 (±2.48), 37.22 (±6.98), and 7.10 (±1.64), respectively. There were no significant differences between the groups (third-year students, fourth-year students, and interns) with respect to knowledge, attitude, and practice (Table 3). This study also revealed that correct responses regarding attitude and practice questions were the highest for questions 5 and 3, respectively, while the lowest correct responses were obtained for questions 6 and 2, respectively.

**Table 3:** Mean (SD) of knowledge, attitude, and practice scores regarding droplet and airborne precautions in subgroups. ^ǂ^Maximum score = 11; ^ǂǂ^Maximum score = 55; ^ǂǂǂ^Maximum score = 11; NS = not significant

Group	Knowledge^ǂ^, mean (SD)	Attitude^ǂǂ^, mean (SD)	Practice^ǂǂǂ^, mean (SD)
Third year	7.56 (2.27)	36.57 (6.41)	6.28 (2.11)
Fourth year	7.70 (2.15)	35.41 (5.80)	6.87 (1.80)
Interns	8.25 (2.36)	37.60 (7.43)	6.98 (1.97)
Total	7.70 (2.48)	37.22 (6.98)	7.1 (1.64)
P-value	0.26^NS^	0.64^NS^	0.73^NS^

We observed a positive correlation between knowledge and attitude (ρ = 0.830, p = 0.041), knowledge and practice (ρ = 0.584, p = 0.028), and attitude and practice (ρ = 0.620, p = 0.036) (Table 4).

**Table 4:** Spearman’s correlation coefficients between knowledge and attitude, knowledge and practice, and attitude and practice scores regarding droplet and airborne precautions stratified by subgroups. * = statistically significant

Group	Knowledge-Attitude	Knowledge-Practice	Attitude-Practice
Third year	0.72	0.14	0.50
Fourth year	0.42	0.32	0.69
Interns	0.17	0.80	0.64
Total	0.830^*^	0.58^*^	0.62^*^

## Discussion

This cross-sectional questionnaire survey was designed to assess the knowledge, attitude, and practice of airborne and droplet isolation precautions among dental students. Our study showed poor compliance with standard isolation precautions among dental students. These results were similar to previously reported studies. The practice of airborne and droplet isolation precautions among Iranian dental professionals and professionals of central India was poor despite good knowledge and attitude [12,16]. Thus, despite sufficient knowledge, it was not being applied in routine dental practice. On the contrary, Jain et al. reported that students showed good knowledge and attitude along with good adherence in practice [13].

This study showed that the mean knowledge, attitude, and practice scores were higher among interns compared to third and fourth-year students (8.25, 47.37, and 37.60, respectively). As the students start handling patients directly in the third year, they get familiar with the protocols over time, which was reflected in the mean scores. In the studies conducted by Askarian et al. and Jain et al., no significant differences in the mean scores were found among the student groups [12,13]. Similar results were reported in previous studies [1,17,18]. On the contrary, in the study by Singh et al, the mean scores for knowledge, attitudes, and practice were 3.75 (1.01), 3.40 (0.75), and 3.35 (1.04) among third-year students, fourth-year students, and interns, and the scores decreasing as the year advanced with lower scores in interns [16].

In response to the COVID-19 pandemic, healthcare professionals around the world have increased their infection prevention and control efforts to limit the spread of the disease, which may explain the similarity in the mean scores for knowledge and practice in this study. A similar study conducted by McCarthy and McDonald in 1997 on general Canadian dentists found that age <40 years, cost of infection control procedures, and lack of concern regarding potential personal risk were some predictors for using infection control precautions [19].

Although a strong positive correlation was observed between attitude and practice, correlations between knowledge and attitude and knowledge and practice were moderately positive. This result emphasizes the significance of continuing education programs to refresh the knowledge of standard guidelines toward infection control practices.

Most students believed in wearing protective equipment, maintaining a safe distance, and isolating the patients with potential risk to prevent the risk of infection. The CDC guidelines recommend, wearing chin-length plastics or surgical masks while treating patients to prevent blood or body fluids from splashing or splattering [20]. This response signifies the positive attitude of dental students toward infection control. A study done by Ramesh and Anuradha among dental professionals in Bangalore and Chennai reported that, despite positive attitudes toward treating patients with infectious diseases, there was a need for more knowledge about infection control [21].

Dental students demonstrated a sufficient level of knowledge, a positive attitude toward infectious control measures, and compliance with these measures. To maintain compliance and update knowledge in accordance with the ever-changing nature of infectious diseases, it is crucial that students receive extensive training on infection control measures.

Study limitations

The method for evaluating the practice might be a limitation of the present investigation. As we were unable to supervise students during their practice, the evaluation of practice was solely based on the respondent’s self-assessment. Thus, it is possible that the responses do not accurately reflect actual knowledge and attitude. Therefore, there may be a disparity between actual and reported levels of practice. Our investigation was limited to dental students from a single dental college and utilized a fixed sample size. Consequently, the study’s power was insufficient to detect statistically significant differences between some of the outcomes.

## Conclusions

In response to the extraordinary circumstances created by COVID-19, healthcare started to modify how they provide care. The present study demonstrates that dental students had adequate knowledge, positive attitudes, and compliance. The upcoming challenge is to create organizational learning by systematically adapting and refreshing the knowledge to achieve long-term change that can improve and sustain infection control practices.

## Appendices

**Table 5:** Study questionnaire.

Statement	Do you agree with the statement?	How much importance should be given to this procedure/protocol	Do you follow this protocol/procedure while treating patients?
Patients with a droplet-spread disease should be isolated in a private room	Yes, No	Very high, High, Intermediate, Low, No importance	Always, Often, Sometimes, Never
Patients with a droplet-spread disease should be kept apart at a distance of at least 150 cm	Yes, No	Very high, High, Intermediate, Low, No importance	Always, Often, Sometimes, Never
Patients with a droplet-spread disease should wear a mask during transport	Yes, No	Very high, High, Intermediate, Low, No importance	Always, Often, Sometimes, Never
Masks should be worn if or when a subject is within a 90 cm distance from a patient under droplet precaution care	Yes, No	Very high, High, Intermediate, Low, No importance	Always, Often, Sometimes, Never
Hospital wards should be notified prior to receiving a patient needing droplet precaution	Yes, No	Very high, High, Intermediate, Low, No importance	Always, Often, Sometimes, Never
Patients with an airborne transmissible disease should be isolated in a private room with negative pressure	Yes, No	Very high, High, Intermediate, Low, No importance	Always, Often, Sometimes, Never
The door of patient’s room with an airborne transmissible disease should always be closed	Yes, No	Very high, High, Intermediate, Low, No importance	Always, Often, Sometimes, Never
Wearing a mask is necessary when entering a room of patients with chickenpox or measles	Yes, No	Very high, High, Intermediate, Low, No importance	Always, Often, Sometimes, Never
All healthcare workers should be vaccinated with the BCG vaccine	Yes, No	Very high, High, Intermediate, Low, No importance	Always, Often, Sometimes, Never
Wards should be notified prior to receiving a patient requiring airborne precautions	Yes, No	Very high, High, Intermediate, Low, No importance	Always, Often, Sometimes, Never
Patients requiring airborne precautions should wear a surgical mask when being transported	Yes, No	Very high, High, Intermediate, Low, No importance	Always, Often, Sometimes, Never
